# Liver fibrosis: concordance analysis between APRI and FIB-4 scores, evolution and predictors in a cohort of HIV patients without HCV and HBV infection

**DOI:** 10.1186/1758-2652-13-S4-P92

**Published:** 2010-11-08

**Authors:** M Mendeni, E Focà, D Gotti, N Ladisa, E Quiros-Roldan, A Vavassori, F Castelnuovo, G Carosi, G Angarano, C Torti

**Affiliations:** 1 Institute for Infectious and Tropical Diseases, University of Brescia, Piazzale Spedali Civili, 1, Brescia, Italy; 2 Institute of Infectious Diseases, Policlinico di Bari, Bari, Italy

## Purpose of the study

Liver fibrosis (LF) progression is fated to become one of the major long-term complications in HIV patients, even in those without HCV or HBV co-infections (HIV-mono-infected). The aim of this study was to assess LF progression in HIV-mono-infected patients and associated risk factors.

## Methods

Observational retrospective study. All HIV naive patients who started HAART from 1996 to 2006 were included. Concordance between FIB-4 and APRI scores was assessed using the weighted kappa coefficient. Rates of transition from lower classes to higher classes were estimated by Kaplan-Meier analysis. Cox regression models were applied to assess possible predictors both at baseline and during the follow-up.

## Summary of results

1,112 naive patients were selected. A moderate concordance between FIB-4 and APRI was demonstrated (K=0.573). For FIB-4, the incidence of transition to higher classes was 0.064 PYFU (95% CI, 0.056-0.072), while for APRI the incidence of transition was 0.099 PYFU (95% CI, 0.089-0.110). Viro-immunological control during HIV infection appeared to reduce the risk of both FIB-4 and APRI transitions. HIV-RNA <500 copies/ml (for FIB-4: HR 2.456 p<0.0001; for APRI: HR 2.084 p<0.0001) and higher CD4 T-cell counts only for FIB-4 (HR 0.881 p=0.0004 for 100 cells higher) during the follow-up were statistically protective. Among baseline variables, for FIB-4 transition, age ≥ 40 years (HR 1.037 p<0.0001) and higher FIB-4 values (HR 1.526 p=0.0038) were associated with increased risk of LF progression, while sexual risk factor for HIV acquisition resulted to be protective (HR 0.524 p=0.0314). For APRI, male gender (HR 1.390 p=0.017), higher GGT values (HR 1.015 p=0.014) and higher APRI values (HR 1.748 p=0.007) were independently associated with APRI transition. A sensitivity analysis demonstrated that DDX drugs (stavudine, didanosine, zalcitabine)as time-dependent covariates were associated with a significant risk of transition with FIB-4 (HR 1.662 p=0.0007) or APRI (HR 1.661 p=0.0001)

## Conclusions

Our data suggest that a better viro-immunological control of HIV infection may slow down fibrosis progression provided that DDX are avoided. Moreover our analysis provided a comprehensive feature of the risk factors that should be controlled in clinical practice

